# Pseudo-outbreak of *Bacillaceae* species associated with poor compliance with blood culture collection recommendations

**DOI:** 10.1007/s10096-024-04925-5

**Published:** 2024-09-27

**Authors:** Alina Maria Borcan, Carmen-Cristina Vasile, Alina-Ioana Popa, Cristina Andreea Badea, Gabriel Adrian Popescu, Daniela Tălăpan

**Affiliations:** 1 Carol Davila University of Medicine and Pharmacy, Bucharest, Romania; 2 National Institute of Infectious Diseases “Matei Balș”, Bucharest, Romania

**Keywords:** *Bacillaceae*, Pseudo-outbreak, Bacteriaemia, Blood culture contamination, Medical cotton wool

## Abstract

**Purpose:**

This study describes a pseudo-outbreak of *Bacillaceae spp.* bloodstream infections that spanned five months starting in May 2023 and the infection prevention measures implemented to control it.

**Methods:**

This retrospective study was conducted at a tertiary infectious disease hospital in Bucharest, Romania. An observational audit of the blood culture collection practice in our hospital was performed, and the materials used during blood culture collection were sampled. Bacterial colonies were identified using MALDI Biotyper. The *Bacillaceae* blood culture positivity rates in the previous four years were compared using the Kruskal‒Wallis rank test.

**Results:**

*Bacillaceae* spp.-positive blood cultures were recovered from 60 patients over a five-month period. In the case of 58 patients, *Bacillaceae* spp.-positive blood cultures were considered contaminated. Two patients were treated for *Bacillus spp*. bacteraemia. The audit revealed significant variation during the preparation of the venipuncture site step and the use of nonsterile medical cotton wool. Medical cotton wool contaminated with species of *Bacillaceae* was found in 10 out of 12 wards. The control measures included repeated training on the blood culture collection procedure and the removal of *Bacillaceae spp.-*contaminated cotton wool.

**Conclusions:**

The pseudo-outbreak was caused by the unjustified use of medical cotton wool for disinfection of the skin and blood culture bottle septums. The investigation of this pseudo-outbreak highlighted a gap in blood culture collection practices at our facility and thus allowed for its improvement.

## Introduction

*Bacillus*,* Brevibacillus*,* Lysinibacillus* and *Paenibacillus* species are aerobic or anaerobic Gram-positive rods ubiquitously found in the environment that produce endospores under aerobic conditions. They belong to the *Bacillaceae* family. When these species are recovered from blood cultures, they are usually dismissed as skin contaminants. However, various species other than *B. anthracis* and *B. cereus* are increasingly recognized for their potential to cause significant human infections [1]. Among the risk factors for opportunistic bloodstream infections caused by these species are immunosuppression [2], hematological malignancies [3], and the presence of a central venous catheter or pacemaker [4, 5]. Oral probiotic administration in immunosuppressed patients has been associated with *B. licheniformis* bloodstream infections [6].

Identification of *Bacillus*,* Brevibacillus*,* Lysinibacillus* and *Paenibacillus* species using matrix-assisted laser desorption/ionization time-of-flight mass spectrometry (MALDI-TOF MS) is hindered by the presence of endospores [7]. The variable vegetative cell-endospore composition of samples directly affects the identification of members of the *Bacillaceae* family [8].

During the first two weeks of May 2023, in a tertiary infectious diseases hospital in Bucharest, Romania, 6 *Bacillaceae-*positive blood cultures were identified, compared with an average of 2.3 per month in the previous 12 months, and the Healthcare-Associated Infection Prevention and Control/Antimicrobial Resistance (HAI/AR) Hospital Committee was alerted.

We report a pseudo-outbreak of *Bacillus*,* Brevibacillus*,* Lysinibacillus* and *Paenibacillus* species caused by the unjustified use of nonsterile medical cotton wool for disinfection of bottle septums and patient’s skin during blood culture collection.

## Methods

A case of *Bacillus* (not *B. anthracis*), *Brevibacillus*,* Lysinibacillus* or *Paenibacillus spp.* laboratory-confirmed bloodstream infection was defined as two or more blood cultures collected from at least two blood draws on the same or consecutive calendar days, and the patient had at least one of the following signs or symptoms: fever (> 38 °C), chills or hypotension with no other recognized cause [9]. The separate blood draws indicate that for each blood culture collected, a separate site preparation (decontamination steps) and a separate venipuncture were performed.

### *Bacillus* spp. isolation and identification

As a routine clinical procedure for blood culture collection, culture bottles (standard aerobic and anaerobic media, fastidious antimicrobial neutralization aerobic and anaerobic media, BioMérieux S.A., Marcy l’Etoile, France) were sent immediately after collection to the microbiology laboratory and incubated at 37 °C. Those identified as positive by the BacT/Alert 3D (BioMérieux, Inc., Durham, NC, USA) were removed and processed as per the laboratory protocol for 24 h, 7 days/week. Cultures were performed on Columbia agar with sheep blood (Thermo Fisher Scientific™-Oxoid, Wesel, Germany) and on lactose agar (CLED, Thermo Fisher Scientific™-Oxoid, Wesel, Germany) and were incubated at 37 °C for 6–18 h. Bacterial colonies were identified using MALDI-TOF MS (MALDI Biotyper^®^, Bruker Daltonics GmbH & KG, Bremen, Germany). The MALDI Biotyper^®^ software version 3.1 was used. A score from 0 to 1.69 indicated no reliable identification, a score from 1.7 to 1.99 indicated probable genus identification, a score from 2 to 2.29 indicated secure genus identification and probable species identification and a score from 2.3 to 3 indicated highly probable species identification.

### Environmental investigation

The Infection Prevention and Control (IPC) Department performed an extensive observational audit of the blood culture collection practices at our hospital. Equipment used during blood culture collection was sampled from all pediatric and adult wards. The samples were inoculated on Columbia Agar with Sheep Blood and on Lactose Agar and incubated for 48 h at 37 °C. Bacterial colonies were identified using a MALDI Biotyper.

Blood culture bottles corresponding to the same production lot as the *Bacillaceae-*positive ones were incubated without prior inoculation in the BacT/Alert 3D system to verify the sterility of the culture media.

### Statistical analysis

Patient demographic and clinical characteristics were summarized using basic descriptive statistics. Patient comorbidities were characterized using the Charlson Comorbidity Index. The data were analyzed using Minitab Statistical Software version 22.1.0. A chi-square test was used to detect differences in the number of contaminated blood cultures corresponding to the period of the pseudo-outbreak in the previous year. The *Bacillaceae* blood culture positivity rate in the previous four years were compared per month and per season using the Kruskal‒Wallis rank test. P values ≤ 0.05 were considered statistically significant.

## Results

**Fig. 1:**
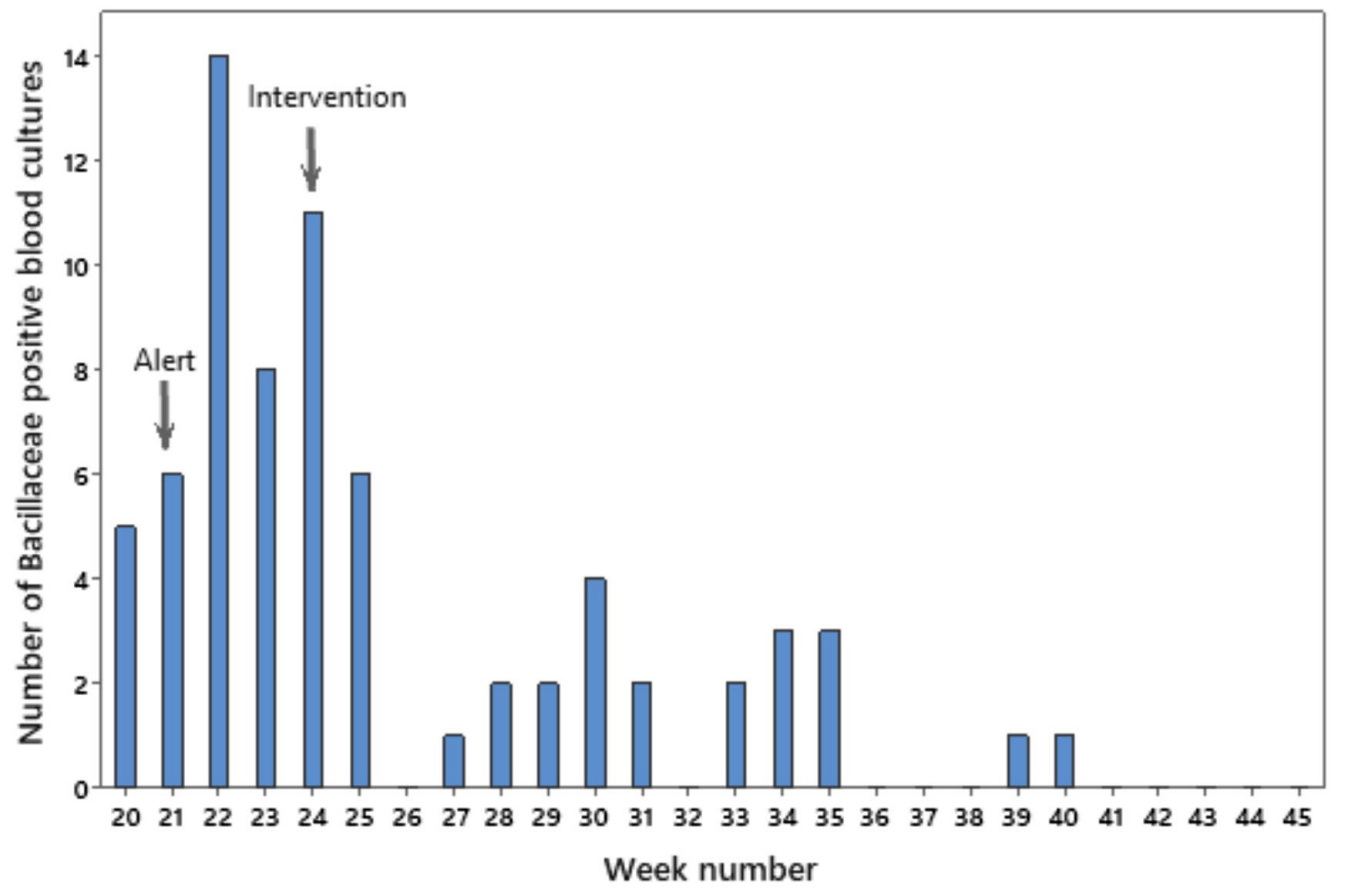
Weekly evolution of the pseudo-outbreak

In total, 71 *Bacillaceae*-positive blood cultures were collected from 60 patients. The first case was identified on May 11, 2023. The peak number of *Bacillaceae*-positive blood cultures was 14 in the third week of the outbreak. Following the implementation of control measures in June, the number of cases decreased, but new cases continued to be detected over a period of 5 months. No additional cases of *Bacillaceae*-contaminated blood cultures were detected in the last quarter of 2023. The weekly number of *Bacillaceae*-positive blood cultures during this pseudo-outbreak is presented in Fig. 1.

**Fig. 2:**
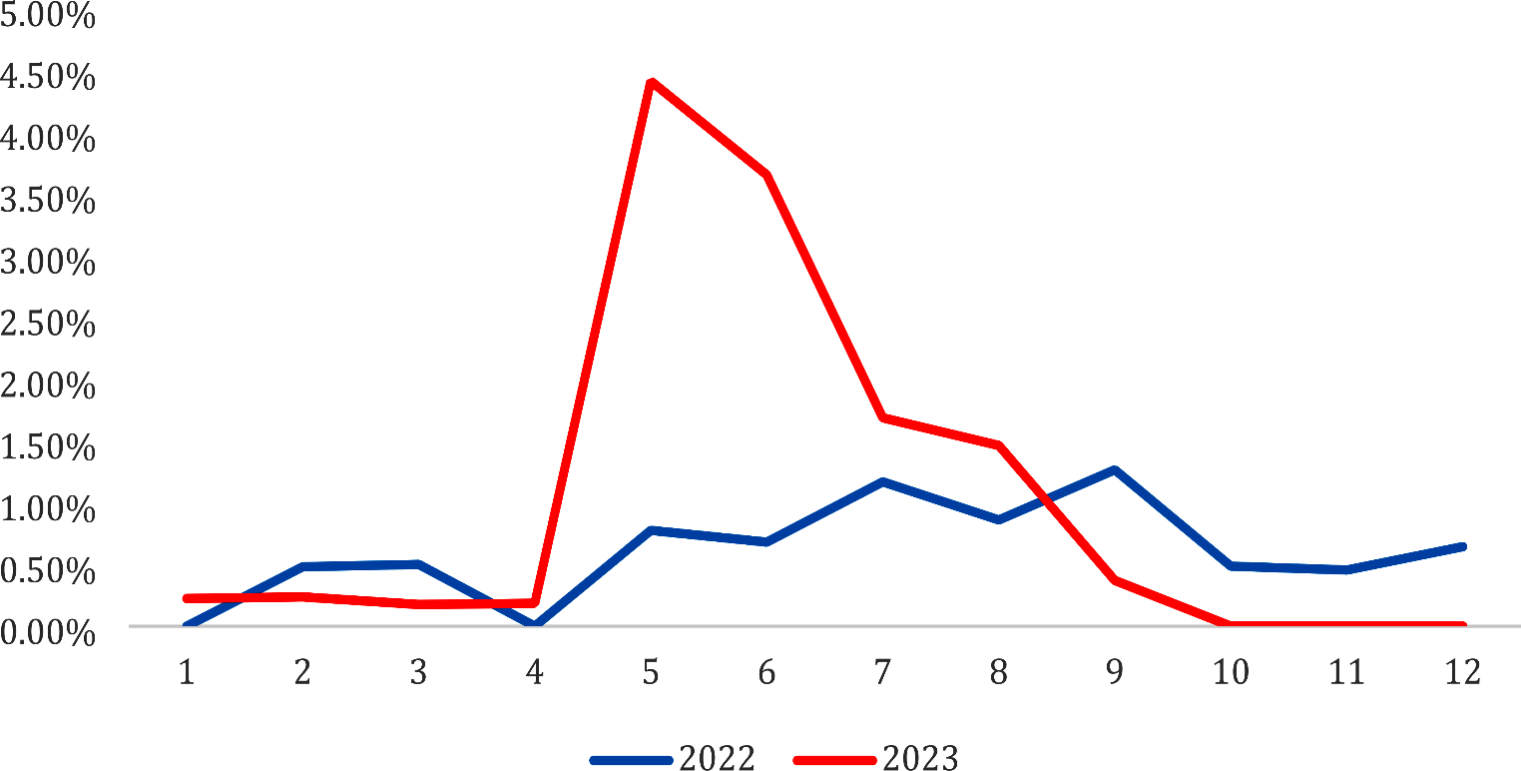
Monthly *Bacillaceae* blood culture contamination rate

The median number of blood cultures processed per month by the microbiology laboratory between May and September 2023 was 575 (IQR = 46, range 540–680). The median monthly contamination rate of blood cultures with species of the *Bacillaceae* family in this period was 1.7% (IQR = 2.2, range = 0.4–4.4%) compared with 0.9% (IQR = 0.4, range = 0.7–1.3%) in the same period in the previous year. A significant increase in positive *Bacillaceae* spp. blood cultures was observed between 2022 and 2023 during this period (*χ²*(1) = 13.168, *p* < 0.001). The monthly percentages of *Bacillaceae*-positive blood cultures collected between 2022 and 2023 are presented in Fig. 2.

The contaminated blood cultures originated from 11 different wards − 4 pediatric wards and 7 adult wards, including the emergency department and intensive care unit.

### Patient characteristics

Overall, the median age was 52.5 years (range of 1–82), and 60% of the patients were male. The most common presentation was fever (41/60), and the majority of patients (58/60) were admitted to inpatient wards. The median number of days since hospital admission to the collection of the first positive blood culture was 0, with an interquartile range of 1 (range 0–42 days).

Eight patients were immunosuppressed, and 6 patients had indwelling invasive medical devices. The patients had an average Charlson Comorbidities Index of 2.1 (range 0–9). The clinical and demographic characteristics of the patients with *Bacillaceae*-positive blood cultures are summarized in Table 1.

**Table 1:** Clinical and demographic characteristics of the patients with *Bacillaceae*-positive blood cultures

Characteristics	All persons (*N* = 60)
Median age (range) - yr	52,5 (1–82)
Male -non./total no.	36/60
Immunosuppression - no./total no.	
Hepatocellular carcinoma	2/60
Hematological malignancy	4/60
Neutropenia	2/60*
Immunosuppressive therapy for renal transplant	1/60
Invasive devices - no/total no.	
Central venous catheter	3/60**
Invasive Mechanical ventilation	1/60
Indwelling urethral catheter	2/60
Cardiac pacemaker	1/60***
Artificial heart valve	1/60
Knee prosthesis	1/60

*One patient had acute leukemia and was neutropenic

**One patient was mechanically ventilated and had a central venous catheter and an indwelling urethral catheter in place

***One patient had a cardiac pacemaker and a prosthetic heart valve

Hematological malignancies included one newly diagnosed multiple myeloma, one acute myeloid leukemia, one newly diagnosed blastic plasmacytoid dendritic cell neoplasm and one chronic lymphocytic leukemia.

Eight patients (11%) had two or more blood cultures positive for the same species of *Bacillaceae* that were collected from at least two blood draws on the same or consecutive calendar days. Six of them presented with fever. One patient was receiving antibiotics at the time of blood collection, which covered the species identified. Given that these patients were not immunocompromised and did not present with any invasive medical devices, other diagnoses were considered, and the blood cultures were dismissed as contaminations.

Two patients received treatment for *Bacillus* spp. bacteraemia without meeting the diagnostic criteria. The first patient was an immunocompromised febrile pediatric patient (15 years old) who was transferred to a hematology unit. The second patient was 42 years old, had multiple invasive devices in place and had been hospitalized in the intensive care unit for a prolonged period of time. The other 58 patients received alternative diagnoses.

### Microbiological characteristics

Of the 71 positive blood cultures, in six of them there were identified two organisms belonging to the *Bacillaceae* family. For 34/77 of the isolates a score of less than 1.69, indicating no reliable identification, was obtained. A score value from 1.7 to 1.99, indicating probable genus identification, was obtained for 23/77 of the isolates. For 13/77 of the isolates a score from 2 to 2.29, indicating secure genus identification and probable species identification, was obtained. Seven of the MALDI Biotyper scores could not be retrieved. The most common genus identified using the MALDI Biotyper among the isolates with a score of more than 1.7 was *Bacillus* (28/36), followed by *Brevibacillus* (6/36), *Paenibacillus* (1/36) and *Lysinibacillus* (1/36). The most common species identified among the isolates with a score of more than 2 were *Bacillus licheniformis* (5/13) and *Brevibacillus borstelensis* (5/13) followed by *Bacillus pumilus* (1/13), *Bacillus thuringiensis* (1/13) and *Paenibacillus glucanolyticus* (1/13). No identification of *Bacillus cereus* was made.

### Environmental samples

The observational audit performed by the IPC team in June 2023 revealed inconsistent blood culture collection practice across all 12 inpatient wards. Significant variation among wards and also among staff from the same ward was observed during the preparation of the venipuncture site. When interviewed, staff from most (10/12) of the wards stated that they used betadine during this step. An alcoholic chlorhexidine skin preparation solution was used in only one-third of the wards. Sanitary alcohol (70% isopropyl alcohol) was also used by medical staff working in all of the wards. Both sterile gauze and medical cotton wool were used for disinfection of the skin and blood culture bottle septums in all of the wards.

A total of 127 materials used during blood collection were cultured from 12 wards; the materials included gloves, chlorhexidine, betadine, 70% isopropyl alcohol, sterile gauze and medical cotton wool. Medical cotton wool contaminated with species of *Bacillaceae* was found in 10 wards. Three out of five batches of medical cotton wool existing in the hospital tested positive. The contaminated medical cotton wool consisted of 50% viscose and 50% cotton. More than half of the packs sampled (15/24) were contaminated. The most common genus identified using the MADLI Biotyper was *Bacillus* (83%), followed by Paenibacillus (10%) and *Brevibacillus* (7%). Multiple organisms belonging to the *Bacillaceae* family were recovered from eight of the medical cotton wool packs. Both sealed (9/12) and unsealed (6/12) packs tested positive.

One unsealed bottle of 70% isopropyl alcohol tested positive out of a total of 23 tested bottles (11 sealed and 12 unsealed). The genus identified using MALDI Biotyper was *Bacillus spp*. None of the isolates had a score of more than 2 indicating probable species identification.

None of the blood culture bottles tested were contaminated.

### Intervention

Three weeks after the initial alert, medical cotton wool belonging to the contaminated lots was removed from all wards. The blood culture collection technique was reinforced by head nurses in each ward according to the local hospital guidelines, which recommended the use of sterile gauze and chlorhexidine in 70% isopropyl alcohol solution for the disinfection of the skin and of the rubber septum of the blood culture bottles. A significant decrease in blood culture contamination with species of *Bacillaceae* was observed after the implementation of control measures (*p* < 0.001). Multiple training sessions were conducted over the following months due to the detection of new clusters despite breaks of 15, 12 and 26 days, which are visible in the epidemic curve. Owing to a shortage of sterile gauze, steam sterilization of medical cotton wool was implemented between July and August 2023.

## Discussion

An increase in *Bacillaceae* spp.-positive blood cultures in our laboratory led to an investigation involving the Microbiology Department, IPC and Infectious Diseases specialists. Initially, in the case of patients who had known risk factors, *Bacillaceae* spp. bacteraemia was considered, but none of these patients met the diagnostic criteria for laboratory-confirmed bloodstream infection. Previous administration of probiotics was considered a possible source of bacteraemia but none of the patients had recently received probiotics containing *Bacillaceae* spp.

An alternative explanation for the increase could have been the seasonal pattern of isolation of *Bacillus* spp. from blood cultures described by Ashkenazi-Hoffnung et al. [10]. The analysis of isolates from blood cultures in our laboratory in the previous four years showed no statistically significant difference between the median percentage of *Bacillaceae*-positive blood cultures per month or per season.

Because most of the patients had no known risk factors for infection with opportunistic pathogens and because the majority of patients had only one positive blood culture, we judged this to be a pseudo-outbreak. The most likely source of this pseudo-outbreak was nonsterile medical cotton wool, which is supported by our findings. First, similar organisms were recovered from the medical cotton wool in 10 out of the 11 wards in which *Bacillaceae* spp. positive blood cultures were identified. Second, the number of positive blood cultures decreased after the removal of contaminated batches of medical cotton wool from the wards. The blood culture bottles were contaminated either by piercing the septum previously disinfected using contaminated cotton wool or from the skin surface previously colonized with *Bacillaceae* species during the stage of skin antisepsis.

Although there appears not to have been any infection in this case, poor compliance with the recommendations for skin antisepsis can lead to serious infections, as reported by Ozkocaman et al. They described an outbreak of 5 cases of hospital-acquired *Bacillus* spp. infections related to the use of nonsterile cotton wool in a hematology unit. The study showed a high morbidity and mortality of *Bacillus* spp. infections in immunocompromised patients. The authors concluded that the isolation of *Bacillus spp*. in blood cultures from patients with hematological malignancy and fever should not be viewed routinely as a contaminant but should be assessed as a potential pathogen; therefore, antibiotic regimens that cover this organism should be considered [3].

The survival of *Bacillus* spp. spores in 70% alcohol has been described previously by Thomas et al. In our case, we considered that the 70% isopropyl alcohol bottle tested positive as a result of cross contamination from the cotton wool packs. The prolonged tolerance of *Bacillus*,* Brevibacillus*,* Lysinibacillus and Paenibacillus* spp. spores to alcohol for more than 12 months, as described by Thomas et al., is concerning because alcohol may act as a source of future contamination [11].

It is difficult to establish whether this is the regular *Bacillaceae* spp. contamination level of cotton wool because comparison of contamination levels is infeasible. There are no published data about quantitative culture of hospital environmental samples, and no standards have been published for acceptable *Bacillus* spp. counts in hospital materials. We hypothesize that the three batches of medical cotton wool we identified had a higher *Bacillaceae spp.* density; hence, the poor practice of blood culture collection in our hospital was revealed. One of the factors that may have influenced the level of contamination was the composition of 50% viscose and 50% cotton in the cotton wool. A study published by Varshney et al. found that out of the six different types of fibers tested, including cotton, bacterial adhesion was maximal for viscose [12].

Outbreaks of *Bacillus* bacteremia associated with contaminated linen have been previously described [13, 14]. Owing to the detection of the *Bacillaceae* spp. in three out of five batches of medical cotton wool in use in the hospital and to the efficacy of the control measures implemented, the environmental sampling was not extended beyond the materials used during blood culture collection.

Nonetheless, our study was limited by the fact that the *Bacillaceae* spp. isolates from blood cultures and from environmental samples were not compared using molecular or genetic sequencing techniques.

In conclusion, this study describes a pseudo-outbreak of *Bacillaceae* spp. bloodstream infection caused by the unjustified use of medical cotton wool during blood culture collection. Errors in medical practice can lead to the real clustering of false infections. It is important to investigate and control pseudo-outbreaks because they can cause unnecessary antibiotic prescriptions, diagnostic procedures, and other potentially harmful interventions to patients [15]. The investigation of this pseudo-outbreak highlighted a gap in blood culture collection practice at our facility and thus allowed for its improvement. We believe that blood culture collection training contributed to the control of the pseudo-outbreak. Therefore, implementing recurrent blood culture collection training in our facility is crucial for reducing the contamination rate of blood cultures and unnecessary antibiotic prescriptions.

## Data Availability

No datasets were generated or analysed during the current study.
